# Muscle fibre type distribution of the thoracolumbar and hindlimb regions of horses: relating fibre type and functional role

**DOI:** 10.1186/1751-0147-56-8

**Published:** 2014-01-27

**Authors:** Heli K Hyytiäinen, Anna K Mykkänen, Anna K Hielm-Björkman, Narelle C Stubbs, Catherine M McGowan

**Affiliations:** 1 Department of Equine and Small Animal Medicine, Faculty of Veterinary Medicine, University of Helsinki, P.O. Box 57, Viikintie 49, 00014, Helsinki, Finland; 2 Mary Anne McPhail Equine Performance Center, Department of Large Animal Clinical Sciences, Michigan State University, College of Veterinary Medicine, Large Animal Clinical Sciences, East Lansing, MI 48824-1314, USA; 3 Institute of Ageing and Chronic Disease, Faculty of Health and Life Sciences, University of Liverpool, Leahurst CH64 7TE, UK

**Keywords:** Muscle fibre type, Postural, Locomotory, Multifidus, Neuromotor control, Physiotherapy

## Abstract

**Background:**

Although the majority of equine muscles have a mixed fibre type distribution indicative of diverse functional roles, the predominance of a fibre type can indicate the primary function of a muscle. The deep epaxial musculature has an important role in core spinal stability in humans, reflected as a predominantly muscle fibre type (MFT) I or postural fibre type. The fibre type of the deep epaxial musculature has not been determined in horses. The objective of the study was to determine the MFT distribution in selected muscles of thoracolumbar and hindlimb region of horses. This included deep epaxial and hypaxial muscles that were hypothesised to have a postural stabilising role. A second objective was to examine differences in MFT distribution between horses bred for endurance (Arabian) and sprinting (Quarter horse). Muscle biopsy samples were obtained from selected thoracolumbar and hind limb muscles of 5 Quarter horses, 4 Arabians, and 2 Thoroughbreds. The myosin heavy chain distribution was determined by gel electrophoresis. Mann–Whitney rank test was used to compare the proportional MFT and differences between breeds.

**Results:**

*Mm. sacrocaudalis dorsalis medialis* and *diaphragm* had the highest proportion of MFT-I. The remaining deep epaxial muscles and the hypaxial muscle *m. psoas minor* had approximately equal MFT I and II proportions. *Mm. psoas major, iliocostalis, longissimus dorsi* and the hind limb muscles contained mostly MFT-IIX. The fibre type distribution was similar between Arabians and Quarter horses, although Quarter horses had more MFT-IIX fibres in *psoas major* (*P* = 0.02) while Arabians had more MFT-I fibres in *m. longissimus dorsi* (*P* = 0.03).

**Conclusions:**

The fibre type distribution of the deep epaxial muscles, *mm psoas minor* and *diaphragm* varied from approximately equal MFT-I and II proportions to predominantly MFT-I suggesting a postural stabilising role possibly important in core spinal stability. In contrast the fibre type proportions of *mm psoas major, iliocostalis, longissimus dorsi* and the hind limb muscles were mainly MFT-II suggesting a locomotory role. Knowledge of fibre type distribution in such a clinically important area can direct diagnosis, prevention and treatment of muscular or neuromotor dysfunction.

## Background

Epaxial muscles of the equine back are situated above the line of the transverse processes and are involved in dorsoventral motion and lateral bending of the spine. The hypaxial muscles are situated below the line of the transverse processes and are involved in flexion and lateral flexion of the spine. In addition to producing movement, morphological and biomechanical studies have suggested that the epaxial and hypaxial muscles play an important role in stabilisation of the equine back and pelvis during both static postures and locomotion [1]. However, the function of the epaxial musculature in different breeds has not yet been clearly defined.

The muscle fibre type (MFT) of skeletal muscle partly defines the function of that muscle [2-4]. Muscles can be categorised functionally as postural (or tonic) and locomotory (or phasic) based on whether they have more MFT-I (slow twitch) or MFT-II (fast twitch) fibres respectively [3]. MFT-I rich muscle is highly oxidative and functions to perform long duration and low energy consumption, postural type work more involved in upholding and maintaining the position rather than the actual movement [5-7]. Muscles with a higher MFT-II proportion are more involved in producing movement or locomotion [6].

The MFT content of a muscle can be determined through electrophoresis and immunostaining [8,9] by separating and identifying the myosin heavy chains (MHC) in the muscle. In the horse immunohistologically studied MHC isoforms can be recognised as MHC type one (I), MHC type two A (IIA), MHC type two X (IIX), or the hybrid MHC-IIA/X. MHC type IIA is more oxidative than type IIX and as such is the more fatigue resistant of the fast twitch MFT [9-11]. Most muscles in horses are a combination of many MFTs, with a predominance of MFT-II [11]. To date, most of the studies in horses have focused on locomotory muscles, in particular, *m. gluteus medius*[9-13].

The type of exercise performed, either due to selection in breeding or as a result of training, can affect muscle fibre type [10,14-18]. The Arabian horse has been bred for long distance endurance-type exercise competing over distances of 40 – 400 km, while the Quarter horse was bred to run a quarter of a mile (400 m) at high speed [19]. A higher proportion of MFT-II than in other breeds has been found in the *m. gluteus medius* of the Quarter horse [12].

The function of the thoracolumbar and lumbopelvic regions during locomotion is complex and requires a coordinated relationship between neural and muscular (motor) activity, which is referred to as neuromotor control [20-22]. In both humans and horses, the muscles involved in dynamic stabilisation have a high MFT-I content and a high number of muscle spindles [23,24] indicative of their complex role in neuromotor control [7]. In the human thoracolumbar region the deepest epaxial muscle *mm. multifidi,* consisting mostly of MFT-I fibres, functions as an antigravity muscle to protect and dynamically stabilise underlying vertebral structures during movement [3,4,20]. The importance of neuromotor control of the back, and its relationship with back pain in humans has been shown in several studies and commented on in review articles [7,21,25,26]. There have been over 145 studies investigating *mm. multifidi* in relation to low back pain in people.

Despite anatomical differences in the orientation and mobility of the equine quadrupedal vertebral column compared to the human, similarities between the anatomy and biomechanical alignment of the deep epaxial muscles of the horse and man has been demonstrated [1,27]. Based on this, it has been hypothesised that the function of the epaxial muscles is comparable to that in humans in that these muscles should provide an essential postural stabilising role of the vertebral column during locomotion. However, determination of the MFT of the musculature of the thoracolumbar region of the horse has been limited to date and would provide important information about the comparative functionality of these muscles. Further, it remains of interest if the tail head muscles, *mm. sacrocaudalis dorsalis lateralis (SCDL)* and *medialis (SCDM)* are functional as well as anatomical extensions of *mm. multifidi* in quadrupeds [1]. However, to the authors’ knowledge, there are no publications involving the equine deep epaxial musculature MFT.

The objectives of this study were to determine the MFT distribution by MHC isoforms in selected thoracolumbar and hind limb muscles in horses and the predominant MFT of each muscle. A further objective was to examine differences in fibre type proportions between the same muscles in Arabian and Quarter horses. We hypothesised that of the muscles studied here, the *mm. multifidi, psoas minor, diaphragm*, *SCDL* and *SCDM* would have a higher proportion of MHC-I, and would therefore be recognised as having a primarily tonic, postural stabilising role. The *mm. gluteus medius, longissimus dorsi, iliocostalis, psoas major* and *biceps femoris* in turn would have a higher proportion of MHC-IIA and MHC-IIX, and hence, would be recognised as phasic, locomotory muscles. It was further hypothesised, that the Arabian would have a higher proportion of highly aerobic MFT-I and MFT-IIA muscles compared to the Quarter horse.

## Methods

Ethical approval was granted by the University of Queensland Animal Ethics Committee. The muscle samples were obtained after euthanasia from 11 healthy adult horses (5 Quarter horses, 4 Arabians, and 2 Thoroughbreds; 8 geldings and 3 mares) aged 4–25 years, destined for the abattoir. The mean age of the Arabians, Thoroughbreds, and Quarter Horses were 13 ± 8 years, 12.5 ± 0.7 years, and 13 ± 7 years, respectively. All horses were of a similar level of fitness and had previously been in light ridden work (Arabians and Quarter horses) or unridden work (walk-trot on horse walker 5 days per week, Thoroughbreds). Samples (n = 154) from 10 muscles were collected: *mm. longissimus dorsi, multifidi, iliocostalis, SCDL, SCDM, psoas major, psoas minor, gluteus medius, biceps femoris,* and *diaphragm*. From the *mm. multifidi*, samples from each of the five fascicles were derived and coded as 1 through 5 from dorsal to ventral, where 1 = most superficial and longest and 5 = deepest and shortest of the fascicles according to Stubbs et al. [1]. Forty of the samples had to be excluded due to sample degradation during transportation.

**Figure 1:**
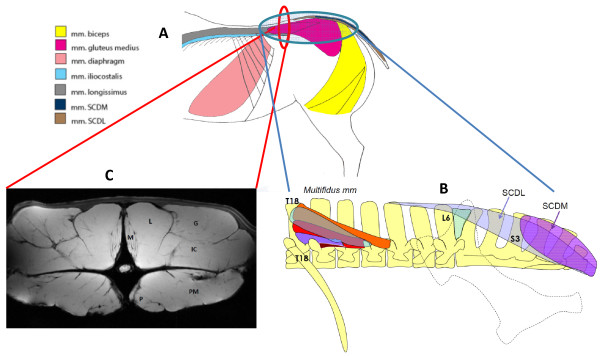
**Illustrations of muscles sampled. A)** A schematic figure presenting *mm. iliocostalis, longissimus dorsi, diaphragm, gluteus medius, biceps femoris, sacrocaudalis dorsalis lateralis (SCDL)* and *sacrocaudalis dorsalis medialis (SDCM).***B)** A schematic diagram depicting; *mm. multifidi* fascicles emanating from the 18th thoracic vertebra crossing between 1–4 vertebral segments attaching caudally onto the mammillary process of the proceeding vertebrae; *m. sacrocaudalis dorsal lateralis* (*SCDL*) with tendinous attachments in the lumbar spine extending onto the sacrum and caudal vertebrae and *m. sacrocaudalis dorsalis medialis* (*SCDM*) emanating from the sacrum and extending to the caudal vertebrae. **C)** Magnetic resonance image (MRI) showing the relationship between epaxial and hypaxial musculature in cross section at the level of L3. Presented are *mm. multifidi* (M), *longissimus* (L), *iliocostalis* (IC), *psoas major* (PM), *psoas minor* (P), *and gluteus medius* (G).

The biopsy samples were taken post mortem from the left, right or both sides of the horse within 2 hours of death, 54 of the samples were collected in duplicate. The side was randomly selected, and the sampling sites are presented in Figure 1. A 5-10 g biopsy was taken from the fleshy portion of the muscle: from the centre of the smaller muscles (e.g. *mm. multifidi* fascicles, *SCDL, SCDM*) or at approximately 5 cm depth at the midpoint of the dissected muscle belly in larger muscles (e.g*. mm. biceps femoris, gluteus medius*). All the samples were obtained by the same person (NCS). The biopsies were cut, rolled in talcum powder, frozen in liquid nitrogen and stored at -80°C until analysis.

### Tissue preparation

Myosin was extracted from 1 cm^3^ of muscle according to Adreani et al. [28] using a Percellys 24 Tissue Homogenizer (Bertin Technologies, Aix en Provence, France). After final centrifugation the supernatant was stored in -80°C until further analysis. The protein concentration was determined with BCA Protein Assay Kit (Thermo Scientific, Rockford, IL, USA).

### Gel electrophoresis

**Figure 2:**
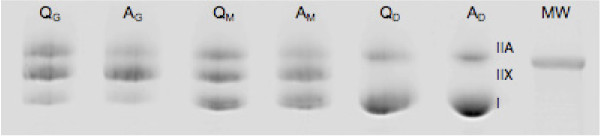
**Gel electrophoresis demonstrating three types of myosin isoforms in *mm. gluteus medius *(**_**G**_**), *multifidi *(**_**M**_**) and *diaphragm *(**_**D**_**) of an Arabian (A) and a Quarter horse (Q).** Molecular weight (MW) 250 kDa.

The samples were incubated for 10 min in a sample buffer (62.5 mM Tris, 10% glycerol, 5% 2-mercaptoethanol, 2.5% SDS, 0.1% bromphenol blue) in room temperature and then heated to 70°C for 10 minutes for denaturation. 1 μg of the sample was loaded into each lane in a modified 10% glycerol PAGE gel and run at 120 V for 28 hours in +4°C. 10 μl of molecular weight marker was added to each gel (Precision Plus Protein Kaleidoscope, BioRad, Hercules, CA, USA). The gels were dyed with Fermentas PageBlue (Fermentas, Vilnus, Lithuania). The gels were scanned (EPSON Expression 1640XL, Seiko Epson Corp, Nagano, Japan) and the intensity of the bands was measured with NIS-Elements software (Nikon Instruments, Melville, NY, USA). Figure 2 shows an example of the gel electrophoresis.

### Immunostaining

To confirm the identity of the bands three MHC antibodies were used: (F36.5B9 (MHC-I) and A4.74 (MHC-IIA) from Alexis Biochemicals (Enzo Life Sciences, Farmingdale, NY, USA) and MY-32 (IIA, IIB, IIX) from AbD Serotec morphoSys UK Ltd (Oxford, UK). The gels were blotted for 18 hours in +4°C after which they were incubated overnight with myosin antibodies in +4°C. The bands were stained with Pierce Super Signal WestDura extended duration substrate (Thermo Scientific, Rockford, IL, USA) and visualised with LAS-3000 (Fuji, Tokyo, Japan) (Figure 2).

### Statistical methods

All data was analysed using SPSS version 18 software (SPSS, Chicago, IL, USA). Results were presented as percentile proportions of overall MFT composition of each muscle. The predominant role of a muscle was established when there was more than 50% of a certain MFT (I or II). When MFT proportions between muscles in all horses were compared, it was noted that only one (IIA) of the three MFT groups was normally distributed, and the two others (I and IIX) were neither normally distributed nor was there equality of variance. The difference in the overall MFT dominance between breeds was established with non-parametric one-way variance analysis, the Mann–Whitney rank test. A Bonferroni adjusted *P* value was used when analysing the statistical significance. Differences in the specific MFT proportions between Arabians and Quarter horses were calculated using the same method. Level of significance was set at *P* < 0.05. Results are presented as mean ± SD as well as mean rank.

## Results

**Figure 3:**
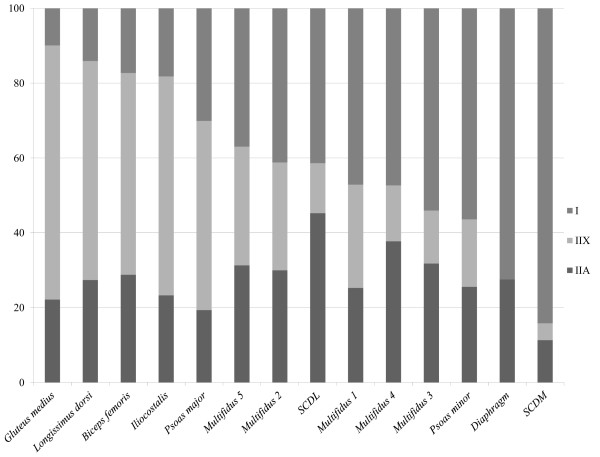
**Muscle fibre type proportions of each muscle in 11 horses (4 Arabians, 5 Quarter horses and 2 Thoroughbreds).***SCDL = m. sacrocaudalis dorsalis lateralis, SCDM = m. sacrocaudalis dorsalis medialis*. Different fascicles of *m. multifidi* are marked with numbers 1 (most superficial) - 5 (deepest).

When the data from all 11 horses were combined, the *mm. SCDM* and *diaphragm* demonstrated a MFT distribution comprised mostly of MFT-I and *m. psoas minor* had 56% MFT-I. The other deep epaxial muscles, including *mm. multifidi* fascicles 1–4 had relatively equal proportions of MFT-I and MFT-II fibres (ranging from 41 – 54%, Table 1). Fascicle 5 of multifidus had almost equal proportions of MFT-I, IIA and IIX. In *SCDL* the amount of MHC-IIA was highest, while in *mm. psoas major, iliocostalis, biceps femoris, gluteus medius* and *longissimus dorsi* the amount of MHC-IIX was highest. Descriptive data is presented in Table 1 and Figure 3.

**Table 1:** Muscle fibre type (MFT-I, IIA, and IIX) percentages (mean ± SD) for each muscle examined in 11 horses (5 Quarter horses, 4 Arabians, and 2 Thoroughbreds); ranked by the MFT-I percentage

**Muscle**	**N**	**MFT-I Mean %**	**MFT-IIA Mean %**	**MFT-IIX Mean %**
*SCDM*_P_	7	84.1 ± 18.4	11.4 ± 10.7	4.5 ± 9.3
*Diaphragm*_P_	8	72.4 ± 12.5	27.6 ± 12.5	0.0 ± 0.0
*Psoas minor*	7	56.4 ± 3.6	25.6 ± 10.5	18.0 ± 12.0
*Multifidus* 3	8	54.1 ± 10.7	31.8 ± 84.7	14.1 ± 8.9
*Multifidus* 1	6	47.1 ± 20.7	25.3 ± 7.9	27.6 ± 8.3
*Multifidus* 4	7	47.3 ± 14.7	37.7 ± 8.4	14.9 ± 11.6
*Multifidus* 2	10	41.2 ± 13.2	30.0 ± 10.1	28.8 ± 19.1
*Multifidus* 5	8	37.0 ± 9.5	31.3 ± 9.1	31.7 ± 14.4
*SCDL*	9	41.4 ± 13.3	45.3 ± 11.6	13.4 ± 12.8
*Psoas major*	10	30.1 ± 10.0	29.4 ± 10.0	50.5 ± 12,8
*Iliocostalis*	9	18.2 ± 7.7	23.3 ± 6.6	58.5 ± 10.7
*Biceps femoris*	9	17.3 ± 6.2	28.8 ± 11.8	53.9 ± 15.4
*Longissimus*	10	14.1 ± 4.2	27.4 ± 5.3	58.5 ± 8.9
*Gluteus medius*	6	10.0 ± 5.1	22.2 ± 6.8	67.9 ± 7.3

Different fascicles of *mm. multifidi* are marked with numbers 1 (most superficial) - 5 (deepest). Muscles considered as postural based on their MFT-I/MFT-II portion are marked with _P_. N = number of samples. *SCDL = m. sacrocaudalis dorsalis lateralis, SCDM = m. sacrocaudalis dorsalis medialis*.

When all muscles studied were pooled, mean MFT-I proportion was 44.9% ± 24.0 in Arabians compared to 38.7% ± 25.1 in Quarter horses; MFT-IIA was 29.3% ± 11.1 in Arabians compared to 22.1% ± 10.1 in Quarter horses; and MFT-IIX was 25.6% ± 21.7 in Arabians compared to 39.3% ± 26.9 in Quarter horses. Descriptive data of muscles per breed are presented in Table 2.

**Table 2:** Muscle fibre type (MFT-I, IIA, and IIX) percentages (mean ± SD) in each muscle examined; comparison between Arabians and Quarter horses

**Muscle**	**Arab/Quarter N**	**Arab MFT-I %**	**Quarter MFT-I %**	**Arab MFT-IIA %**	**Quarter MFT-IIA %**	**Arab MFT-IIX %**	**Quarter MFT-IIX %**
*SCDM* A_P_, Q_P_	1/4	86.7	82.8 ± 25.9	13.3	9.3 ± 14.7	0.0	7.9 ± 11.7
*Diaphragm* A_P_, Q_P_	3/5	80.4 ± 9.4	67.6 ± 12.3	19.6 ± 9.4	32.4 ± 12.3	0.0 ± 0.0	0.0 ± 0.0
*Multifidus* 4, A_P_	3/2	59.3 ± 14.9	37.8 ± 1.9	33.1 ± 5.8	34.8 ± 7.3	7.6 ± 10.7	27.4 ± 9.3
*Psoas minor* A_P_, Q_P_	3/4	58.6 ± 4.9	54.8 ± 1.3	26.2 ± 13.3	25.1 ± 10.1	15.3 ± 15.0	20.1 ± 11.2
*Multifidus* 3 A_P_, Q_P_	3/3	53.5 ± 16.7	57.8 ± 8.1	33.0 ± 1.8	26.9 ± 2.2	13.5 ± 14.9	15.4 ± 6.8
*Multifidus* 2, A_P_	3/5	52.4 ± 12.1	38.3 ± 12.4	30.6 ± 4.1	24.5 ± 9.8	17.0 ± 15.0	37.1 ± 21.5
Multifidus 1	3/1	51.4 ± 30.8	42.1 ± 12.1	24.1 ± 9.1	22.4 ± 6.1	24.5 ± 23.9	35.5 ± 18.2
*SCDL*	3/4	39.1 ± 5.2	45.9 ± 19.2	54.9 ± 11.5	36.5 ± 8.1	6.0 ± 6.3	17.6 ± 16.5
*Multifidus* 5	3/3	37.2 ± 7.5	36.3 ± 15.8	35.3 ± 4.4	22.4 ± 6.4	27.4 ± 11.5	41.3 ± 18.9
*Psoas major*	4/5	36.6 ± 6.2	26.2 ± 11.2	21.5 ± 5.2	13.0 ± 3.7	41.9* ± 3.2	60.8* ± 9.1
*Biceps femoris*	2/5	20.0 ± 0.1	15.6 ± 7.7	42.6 ± 9.0	20.3 ± 2.3	37.4 ± 9.1	64.1 ± 7.5
*Iliocostalis*	4/3	19.5 ± 10.4	14.0 ± 5.2	25.8 ± 4.2	16.0 ± 0.9	54.7 ± 8.1	70.0 ± 4.7
*Longissimus*	4/4	16.9* ± 4.6	9.9* ± 1.4	29.8 ± 4.9	23.7 ± 5.4	53.2 ± 8.0	66.4 ± 5.8
*Gluteus medius*	3/3	12.2 ± 6.3	7.6 ± 3.2	25.6 ± 8.7	18.8 ± 2.0	62.2 ± 2.5	73.6 ± 5.2

Different fascicles of *mm. multifidi* marked with numbers 1(most superficial) - 5 (deepest). Significant differences between breeds (*) were calculated according to the Mann–Whitney rank test. Muscles considered as postural based on their MFT-I/MFT-II portion in Arabian (A_P_) and Quarter horse (Q_P_). N = number of horses. *SCDL = m. sacrocaudalis dorsalis lateralis, SCDM = m. sacrocaudalis dorsalis medialis*.

When the distribution of MFT’s in specific muscles were compared between Arabians (sample n = 42) and Quarter horses (sample n = 51), a significant difference between breeds was seen in two muscles; *mm. longissimus,* and *psoas major* (Table 2). The distribution of MFT-IIX in the *m. psoas major* was lower in the Arabians (mean rank 2.5) than in Quarter horses (mean rank 7, *P* = 0.02). The distribution of MFT-I in the *m. longissimus* was higher in Arabians significantly (mean rank 6.5) than Quarter horses (mean rank 2.5, *P* = 0.03).

## Discussion

A fibre type distribution typical of a predominantly postural stabilising function was found in the *diaphragm* and *SCDM* but the rest of the deep epaxial and hypaxial muscles only had approximately equal proportions of MFT I and II indicating dual postural and locomotory roles. As expected, the superficial epaxial and hind limb muscles studied had a fibre type distribution indicative of a primarily locomotory function. Based on the previous studies on humans it is possible to assume that with such high MFT-I content, *SCDM* could be rich in muscle spindles, and would therefore also have a proprioceptive function [23,24]. However, muscle spindles were not analysed in this study.

The dominance of MFT-II in *SCDL* was unexpected. The structure of the *SCDL* and the more superficial fascicles of *mm. multifidi* are quite similar, as are the structures between the *SCDM* and the deeper and shorter fascicles of the *mm. multifidi*[1]*.* The *SCDL* is longer and more superficial than the *SCDM*, originating from L4-6, crossing the lumbosacral junction and several coccygeal vertebrae. In comparison, the *SCDM*, is short, originating from S3. *SCDL* has long tendinous attachments and a bursa, which both are indicative of notable motion and force associated with the muscle [1]. Therefore, the difference between the *SCDL* and *SCDM* MFT might be related to the length of the fascicles. However, while the *SCDL* and *SCDM* anatomically appear to extend the *mm. multifidi*[1], they also have the function of tail movement [29] which may explain the MFT distribution. Further, the most prominent MFT was the highly oxidative MFT-IIA and not the IIX, as in all other locomotory muscles studied here. As type IIA muscles are fast twitch and fatigue resistant, this may represent the dual functions of *SCDL*; both tail movements and neuromotor stabilisation of the lumbosacral and caudal vertebral regions.

In deviation from our hypothesis was the mixed MFT of the *mm. multifidi*. Only the 3^rd^ out of the five fascicles contained over 50% MFT-I when all breeds were examined as one group. In Arabians, all fascicles contained over 50% MFT-I. The proportion of MFT-I in the *mm. multifidi* was comparable but slightly lower overall across horse breeds (37-54%) than has been reported in cadaver samples from lumbar regions of healthy humans (43-69%) [2-4]. However, when surgical specimens and those from people with a history of lower back pain were included, the proportion of MFT I in people varied widely from 36%-97% [4,30]. This may imply that some of the horses in the current study had an unknown history of back pain, or it might be due to the difference in the biomechanics (and reduced vertebral mobility) and/or the locomotory behavior between humans and horses [1]. The demands of the equine back musculature through mechanical pressure and forces when ridden may present different challenges to the musculature, potentially explaining the requirement for a mixed MFT [31,32]. Further studies are warranted to develop these findings.

The proportion of MFT-I fibres differed between fascicles and no clear pattern of MFT and fascicle length or depth was seen in horses in the current study. A balance between MFTs is needed to uphold optimal posture in bipeds and to ensure the functionality and support against both sudden and long duration physical demands [33]. In humans, *mm. multifidi* stabilised the vertebral column during arm movements, acting in anticipation of movement with the more superficial fibres being direction specific and the deeper fibres were non-direction specific [22]. The resultant hypothesis from this work was that the superficial fibres contribute to the control of spinal orientation while the deeper fibres control intersegmental motion [4,22]. The results of the present study show that this division may not be as simple as superficial and deep fibres, at least not in horses.

The *mm. diaphragm* and *psoas minor* were also found to have a high proportion of MFT-I. Even though slightly differing in percentage values, similar proportions of MFTs within the *m. diaphragm* were seen in two previous studies on Thoroughbred horses [11,13]. The authors were unable to find any previous studies of either human or veterinary medicine with regards to the *m. psoas minor* MFTs*. M. psoas minor* is present in only 50% of humans [34]. Whereas the function and relevance of the muscle is considered minor in humans, corresponding information on horses was not found. As *m. psoas minor* is not a prime mover in humans, but merely a weak continuum of *m. psoas major* and a part of the *m. iliopsoas* system, it may provide more postural support when present. Based on our findings of its high MFT-I proportion in the horse, *m. psoas major* may also provide a proprioceptive input to the vertebral column.

For the locomotory muscles, our findings were very similar to previous research in Thoroughbreds in respect to the MFT-I:II fibre type ratio [11,13]. However, in the present study we found a higher MFT-IIX proportion of the MFT-II fibres. Training affects the MFT proportions of the muscle, with an increase in the MFT-IIA:IIX ratio shown in different breeds [10,35-37]. Therefore, the lower IIA:IIX ratios in these horses compared to the Thoroughbred racehorses was likely to be due to the reduced training experienced by the horses in the present study, as they had only had light exercise prior to euthanasia. Differences could also be due to different sampling depth, with the equine *m. gluteus medius* shown to have more MFT- I fibres in its deeper portions and more MFT- II fibres in the more superficial layers [9,10]. However, where variations existed, it was in the MFT- IIA:IIX ratio and not in the type I:II ratio and so this is less likely to be a factor when comparing the present study to the work of Kawai et al. [11,13]. However, it would have been interesting to have more data on the horses analysed in this study, e.g. what type of sports they had been trained for and for how many years.

The MFT of the equine *m. iliocostalis* has not been previously reported. It has been reported to stabilise lumbar vertebra and ribs, extend and laterally flex the vertebral column in addition to it possibly functioning during expiration in the horse [38]. In our study *m. iliocostalis* was found to clearly belong to the locomotory group of muscles: especially in the Quarter horse with a MFT-IIX proportion of 70%.

Knowledge of fibre type distribution in such clinically important areas as the thoracolumbar and hindlimb regions can direct diagnosis, prevention and treatment of muscular or neuromotor dysfunction. For example, when selecting a muscle to biopsy for neuromuscular disease or when prescribing physiotherapeutic exercises to rehabilitate the muscles of the horses’ back [39]. Based on the results of the present study, exercises could be targeted based on MFT, specifically altering amount, speed, and resistance of the exercises used.

Despite some breed differences being present between MFT of Arabians and Quarter horses, the major classification of muscles according to their functional roles was not affected by breed and the same general pattern with all horses was seen. Even though neither a comparison between these two breeds, nor all the same muscles have been studied previously, related publications do exist, showing differences between breeds. One study compared the MFT proportions in *mm. biceps femoris and triceps* in Belgian, Standardbred, Thoroughbred, Quarter horse and Welsh breed fillies. The study showed that there was a significant variance in MFTs between breeds: Thoroughbreds having a predominance of red (MFT-IIA) and Belgians of white (MFT-IIB) muscle [14]. Another study comparing the effects of training on the MFT proportions in the *m. gluteus medius* muscle of Andalusian, Arabian and Thoroughbred horses, found differences in MFT proportion between the breeds [40]. In our study Arabians tended to have more highly oxidative muscle fibres than Quarter horses overall, with Arabians having more MFT- IIA in *m. psoas major* and a higher proportion of MFT-I in *m. longissimus*.

There were, however, several possibilities for error during sample collection that may have affected our results [41]. Not only the method of sampling, the impact of analysing different number of samples from each horse, but also the heterogeneity of muscle tissue may have had an impact on the results. In human studies side differences within a subject have been shown to be present. For example, *m. psoas major* has been shown to have a different MFT proportion on the subjects’ left and right side [42]. In our study, samples were collected from only one side of each horse, and the side was randomly selected. Getting samples only from one side may have affected the results to some extent, but this bias was minimised by randomly selecting the side. Another weakness of the study is the limited sample size. Further studies with larger sample size and more bilateral samples are warranted.

## Conclusion

Despite the limited sample size the results are novel and interesting. The fibre type distribution of the deep epaxial muscles and *mm. psoas minor* varied from approximately equal type I and II fibre type proportions to predominantly type I suggesting a postural stabilising role possibly important in core spinal stability. In contrast the fibre type proportions of *mm. psoas major, iliocostalis, longissimus dorsi* and the hind limb muscles were mainly type II suggesting a locomotory role. In this study there were limited differences between breeds despite the work the horses were bred to perform and the same general pattern for major classification of muscles according to their functional roles was seen. More studies on the differences between postural and locomotory muscles in horses and different variables associated with them (breed, pain, therapeutic exercises) are needed to confirm the findings of our study, and to gain more understanding of their nature and function.

## Abbreviations

MFT: Muscle fibre type; MHC: Myosin heavy chain; SCDL: *Musculus sacrocaudalis dorsalis lateralis*; SCDM: *Musculus sacrocaudalis dorsalis medialis.*

## Competing interests

The authors declare that they have no competing interests. No external funding was received.

## Authors’ contributions

HKH performed the statistics and drafted the manuscript. AKM carried out the sample analysis and data collection in addition to contributing to the manuscript. NCS participated in study concept, sample collection and participated in drafting the manuscript. AKH-B contributed to the manuscript. CMMG participated in study concept, study design, sample collection and drafting the study. All authors read and approved the final manuscript.
